# Natural Products for Ameliorating Degenerative Diseases

**DOI:** 10.1155/2018/3757403

**Published:** 2018-06-28

**Authors:** Eman Al-Sayed, Indumathi Somasundaram, Dinesh Dhamecha, Hanan Hagar

**Affiliations:** ^1^Department of Pharmacognosy, Faculty of Pharmacy, Ain-Shams University, Cairo 11566, Egypt; ^2^Department of Stem Cell and Regenerative Medicine, D. Y. Patil University, Kolhapur, Maharashtra 600 044, India; ^3^Department of Stem Cell and Regenerative Medicine, National Institute of Nutrition, Secunderabad, Andhra Pradesh, India; ^4^Basic Science Research Center, KLE University, Nehru Nagar, Belgaum, Karnataka 590 010, India; ^5^Pharmacology Unit (31), Medical College, King Saud University and King Khalid University Hospital, Riyadh, Saudi Arabia

Herbal medicines have been used for thousands of years for the treatment of several ailments. Herbal medicines contain different classes of secondary metabolites with beneficial health effects, which are considered as important sources of lead structures. Recently, there has been a resurgence of interest in the use of natural products because of their reduced side effects compared to synthetic drugs. Several synthetic drugs have been used for the treatment of different metabolic and degenerative diseases. However, some of these drugs are expensive and produce serious adverse effects. The use of these drugs may be unsuccessful in some cases due to development of tolerance and relapses and, therefore, may be of limited therapeutic benefits. In this regard, natural products represent an important source of new lead structures for the development of many drugs for ameliorating oxidative stress and metabolic and degenerative diseases. This special issue was dedicated to integrating the pharmacological mechanisms of natural products in ameliorating oxidative stress-associated diseases and the chemistry of secondary metabolites. The editors would like to thank all authors who have contributed their original research articles and reviews to this special issue. Thirteen articles were published in this issue. The topics included in this issue comprised three major categories:

- Natural products with neuroprotective potential
- Toxicology studies of natural products on hepatotoxicity and effect of natural products on oxidative stress related diseases
- Effect of natural products on endocrine diseases, metabolic syndrome, and osteoporosis

The first category included a study by D.-D. Mao et al. who highlighted the potential for treatment of Alzheimer's disease by a traditional Chinese medicine, Qingxin Kaiqiao Fang (QKF), through the modulation of hippocampus mRNA expression of inflammation-related genes IL-1ß, GFAP, and amyloid-beta (A*β*) in an Alzheimer's disease rat model. From this study, it was evident that QKF significantly modulated the expression of these parameters; therefore, the authors have concluded that QKF has a therapeutic potential against Alzheimer's disease and can be used for ameliorating age-associated disorders. Vitamin E has been shown to possess potent antioxidant and neuroprotection activities. A. Abd Jalil et al. described the effect of a tocotrienols-rich fraction (TRF) from palm oil and alpha-tocopherol (*α*-TCP) in modulating the glutamate receptor and neuron injury markers in an oxidative stress model of neural cells derived from embryonic stem cell cultures. To elucidate the mechanism of action, the authors have used an in vitro model of oxidative stress in neural-derived embryonic stem (ES) cell cultures. TRF and *α*-TCP exerted protective and antioxidant effects against glutamate toxicity in neural-derived ES cells. Therefore, it was recommended that these treatments could be developed into potential therapeutic agents for Alzheimer's disease. A standardized Chinese herbal formula, Xiao-Er-An-Shen, was proven to regulate neuron differentiation and antioxidant activity by Z. Li et al. The decoction prepared from this herbal formula was shown to prevent the impairment of neurogenesis under oxidative stress and to exert neuron protection by modulation of differentiation and antioxidant activity in cultured PC12 cells. H. Wang et al. examined the effect of* Ficus deltoidea* (FD) aqueous extracts on lipopolysaccharide-induced TNF-a production from microglia. The authors have shown that the extract has a therapeutic potential against neuroinflammatory diseases. This was evident from its effect on the release of tumor necrosis factor-alpha (TNF-*ɑ*), the expression of CD40, and the morphology of microglial cells in lipopolysaccharide- (LPS-) activated BV2 cells. An article by Y. Fu et al. has demonstrated the potential therapeutic role of* trans*-cinnamaldehyde for ameliorating neuroinflammation-mediated neurodegenerative diseases in BV2 microglial cells with lipopolysaccharide stimulation. TCA pretreatment significantly inhibited LPS-induced production of NO and expression of iNOS, COX-2, and IL-1*β* and normalized the morphological changes in BV2 cells. TCA markedly attenuated microglial activation and neuroinflammation by blocking nuclear factor kappa B (NF-*κ*B) signaling pathway. It was concluded that TCA greatly reduced LPS-elicited neuronal death and exerted neuroprotective effects.

Toxicology studies of natural products on hepatotoxicity and oxidative stress comprised an article by M. S. Hossen et al. who highlighted the hepatoprotective effect of coadministration of two mushroom extracts from* Ganoderma lucidum *(GL) and* Auricularia polytricha *(AP) against carbofuran- (CF-) induced toxicity in rats. Emodin, the major active component of* Rheum officinale* and* Polygonum cuspidatum*, has been reported to have antifibrotic effect. It was evident from the work done by X.-A. Zhao et al. that emodin can alleviate the degree of liver fibrosis induced by carbon tetrachloride by reducing infiltration of Gr1^hi^ monocytes. Emodin significantly inhibited hepatic expression of interleukin-1*β* (IL-1*β*), tumor necrosis factor-alpha (TNF)-*α*, transforming growth factor-*β*1 (TGF-*β*1), granulin (GRN), and monocyte chemoattractant protein 1 (MCP-1). These results suggest that emodin is a promising candidate in the prevention and treatment of liver fibrosis. H. Peiyuan et al. have emphasized that resveratrol alleviated alcoholic liver disease (ALD) through the regulation of oxidative stress, apoptosis, and inflammation. Resveratrol significantly attenuated alcohol-induced elevation of liver enzymes and improved hepatic antioxidant enzymes. Resveratrol also attenuated alcohol-induced CYP2E1 increase, oxidative stress, and apoptosis. The data suggests that resveratrol is a promising natural therapeutic agent against chronic ALD.

Effect of natural products on endocrine pharmacology, metabolic syndrome, and osteoporosis included the study by N. Rivera-Yañez et al. on the hypoglycemic and antioxidant effects of propolis of Chihuahua in a model of experimental diabetes. This investigation demonstrated that propolis possessed hypoglycemic and antioxidant activities and can alleviate symptoms of diabetes mellitus in mice. The ethanolic extract of propolis of Chihuahua significantly inhibited the increase in blood glucose and the loss of body weight in diabetic mice. EEPCh increased plasma insulin levels in STZ-diabetic mice, whereas in untreated diabetic mice there was no detection of insulin. The findings of an article by M. Y. Ali et al. suggested that* Garcinia pedunculata* fruit methanol extract is effective against hyperglycemia and may be used in the treatment of diabetes. In addition, the extract ameliorated diabetes complications and overall oxidative stress-mediated pathological conditions. Furthermore, M. R. Oumarou et al. proved that* Lannea acida* A. Rich. (Anacardiaceae) ethanol extract exhibits estrogenic effects and prevents bone loss in an ovariectomized rat model of osteoporosis. The authors highlighted the estrogenic activity of* Lannea acida* barks ethanolic extract and its antiosteoporotic potential in ovariectomized Wistar rats.* Lannea acida* extract improved bone microarchitecture and could restore normal bone mineralization by increasing the inorganic phosphorus and calcium level in bone. These findings provide evidence that* Lannea acida* is a potential alternative for the prevention of postmenopausal osteoporosis. The study by J. Huang et al. demonstrated that icariin from Herba Epimedii, a widely used traditional Chinese medicine, exerted antiosteoporosis activity by regulating bidirectional differentiation of bone marrow mesenchymal stem cells (BMMSCs) through canonical Wnt signaling pathway. Fat infiltration within bone marrow is easily observed in some postmenopausal women. The main origin of this fat derives from bone marrow mesenchymal stem cells (BMMSCs). The increment of adipocytes derived from BMMSCs leads to decreased osteoblast derived from BMMSCs, so the bidirectional differentiation of BMMSCs is a significant factor contributing to osteoporosis. A review by S. Sameh et al. focused on the genus* Spondias*, which is widely used in traditional medicine for the treatment of many diseases. The authors compiled many pharmacological and phytochemical reports on* Spondias*. The authors highlighted that, in view of the limited drugs available for treating degenerative diseases, natural products from* Spondias* plants represent a promising therapeutic strategy in the search for new and effective candidates for treating degenerative diseases.

*Eman Al-Sayed* *Indumathi Somasundaram* *Dinesh Dhamecha* *Hanan Hagar*

